# Decreased chromobox homologue 7 expression is associated with epithelial–mesenchymal transition and poor prognosis in cervical cancer

**DOI:** 10.1515/med-2021-0015

**Published:** 2021-03-09

**Authors:** Ping Tian, Chen Zhang, Cailing Ma, Lu Ding, Ning Tao, Li Ning, Yan Wang, Xianting Yong, Qi Yan, Xin Lin, Jing Wang, Rong Li

**Affiliations:** College of Public Health, Xinjiang Medical University, No. 393, Xinyi Road, Urumqi 830054, Xinjiang, China; The Fifth Affiliated Hospital, Xinjiang Medical University, Urumqi 830011, Xinjiang, China; State Key Laboratory of Pathogenesis, Prevention and Treatment of High Incidence Diseases in Central Asia (PPTHIDCA), Xinjiang Medical University, Urumqi 830054, Xinjiang, China; Department of Gynecology, The First Affiliated Hospital, Urumqi, Xinjiang Medical University, Urumqi 830054, Xinjiang, China; Tumor Hospital Affiliated to Xinjiang Medical University, Urumqi 830011, Xinjiang, China; Postdoctoral Research Center on Clinical Medicine, The First Affiliated Hospital, Xinjiang Medical University, Urumqi 830054, Xinjiang, China

**Keywords:** cervical cancer, chromobox homologue 7, epithelia–mesenchymal transition, E-cadherin, prognosis

## Abstract

The aim of this study was to evaluate the association of the chromobox homologue 7 (CBX7) expression with the epithelial–mesenchymal transition in cervical cancer (CC), as well as with the disease prognosis. CBX7, E-cadherin (E-cad), and vimentin (VIM) expression levels were detected with immunohistochemistry. The relationship between the expression of CBX7, E-cad, and VIM expression and conventional clinicopathological characteristics of CC were evaluated. The positive expression rates of CBX7 and E-cad in the CC tissues were lower than the adjacent non-tumorous cervical tissues. Moreover, the VIM expression level was higher. The CBX7 expression was positively correlated with the E-cad expression, whereas was negatively correlated with the VIM expression. Furthermore, CBX7 was associated with the disease clinical staging, histological differentiation, lymph node metastasis, and vascular invasion. Patients with negative CBX7 expression showed decreased overall survival rates compared with those with low or high CBX7 expression. Multivariate Cox regression analysis indicated that the decreased CBX7 expression was an independent predictor for the poor prognosis of CC. In conclusion, the absence of CBX7 is associated with the histologic differentiation, lymphatic metastasis, vascular invasion, and poor prognosis of CC. CBX7 may be an independent prognostic factor for the prognosis of CC patients.

## 1 Introduction

Cervical cancer (CC) is one of the most common gynecological malignant diseases, and the fourth most frequent cancer in the world [1,2]. Although the mortality of CC has declined in recent years, there are more than 5,00,000 new cases of CC, and CC causes more than 2,50,000 deaths each year. The CC morbidity and the total death cases continue to increase [1], and the 5-year survival rate of CC is only 68% [3], with the tumor metastasis as one of the main causes for death. Therefore, for better prognostics, it is of great significance to develop new therapeutic strategies for CC and investigate the molecular and biologic mechanisms of tumorigenesis.

Previous studies have shown that chromobox homologue 7 (CBX7) is ubiquitously expressed in glioma, endometrium, and other diseases, which may play a role in anticarcinogenesis. The expression levels of CBX7 are reduced in some tumors, and it is correlated with the clinicopathological parameters of several tumors, including breast cancer, bladder cancer, colon cancer, and glioma [4,5,6,7]. On the other hand, CBX7 has been described as a carcinogen in ovarian cancer, gastric cancer, and lymphoma [8,9,10]. However, the role of CBX7 in the pathogenesis and development of CC has not yet been clearly defined.

Epithelial–mesenchymal transition (EMT) has been characterized by the morphological changes, from the cuboidal to spindle-shaped phenotype. Concerning the associated biochemical changes, EMT has been defined by the loss of epithelial markers (such as E-cadherin [E-cad]) and has been characterized by the overexpression of nominal mesenchymal elements (such as vimentin [VIM]). Moreover, the EMT has been related to the migration and invasion capabilities, improving the mesenchymal phenotypes and motility. Studies have found that numerous regulatory factors and the tumor microenvironment are involved in the occurrence of EMT [11,12]. Bao et al. [13] have shown that the occurrence and development of glioma is related to the occurrence of EMT promoted by CBX7 [13]. However, in the pathogenesis and development of CC, whether CBX7 is related to the EMT occurrence needs further verification.

In this study, the CC tissues were collected, and the expression levels of CBX7 and EMT markers were investigated with the immunohistochemistry (IHC) staining. The significance of CBX7 expression in relation to the clinicopathological features and disease prognosis of CC was also analyzed.

## 2 Methods

### 2.1 Study subjects and tissue samples

Totally, 120 patients with primary CC (stage I, stage II, and stages III–IV) were included in this study, with the mean age of 52.9 ± 7.6 years (ranging from 25 to 67 years), who underwent the surgical treatment at the First Affiliated Hospital of the Xinjiang Medical University, from January 2011 to December 2013. The formalin-fixed and paraffin-embedded tumor samples were obtained during electronic colposcopy, cervical conization, and hysterectomy. The matching adjacent non-tumorous tissues were collected and used as control specimens. All patients were primarily treated with optimal surgery by skilled surgeons in the gynecologic oncology. Tumor staging was performed based on the International Federation of Gynaecology and Obstetrics (FIGO) classification, which was reviewed by two expert gynecologists. The histologic diagnosis was reviewed by experts in the gynecological pathology. Written informed consent was obtained from every patient, and the study was approved by the ethics review board of the Xinjiang Medical University.

All these CC patients received the standard adjuvant chemotherapy after operation, with the available clinical outcomes. These patients had been followed up for 5 years. The survival time was from 10 to 60 months, with the mean survival period of 49.64 months. Follow-up information included overall survival (OS), which was calculated from the confirmed diagnosis to the date of death, or until the end of follow-up period.

### 2.2 IHC staining

The expression levels of CBX7 and E-cad in the tissues were determined by the IHC, using a highly sensitive streptavidin–biotin–peroxidase detection kit (PV6001; ZSGB-BIO, Beijing, China). The tissue was paraffin-embedded, which was cut into 4 µm serial sections. The sections were incubated in 3% H_2_O_2_ (1,301; Dexin Kang, Dezhou, Shandong, China) at room temperature for 20 min, and the antigen retrieval was performed with 0.01 M citrate buffer at 95°C for 10 min. After blocking with normal sheep serum at room temperature for 30 min, the section was incubated by the anti-CBX7 (1:200 dilution; ab21873; Abcam, Cambridge, UK), anti-E-cad (1:500 dilution; ab40772; Abcam) and anti-VIM (1:300 dilution; ab92547; Abcam) monoclonal antibodies, respectively, at 4°C overnight. Then the section was incubated with HRP-conjugated anti-rabbit En Vision system (PV-6001) at 37°C for 20 min, followed by staining with diaminobenzidine hydrochloride (DAB; ZLI-9618; ZSGB-BIO). Then the sections were counterstained with hematoxylin and observed under microscope.

### 2.3 Evaluation and quantification of immunostaining

Positive IHC staining of CBX7, E-cad, and VIM was represented by brown–yellow granules in the nucleus, cell membrane, and cytoplasm, respectively. To quantify the protein expression levels, both the intensity and immunoreactivity were evaluated and scored, by two pathologists independently. The IHC intensity was scored as follows: 0, negative staining; 1, weak staining; 2, moderate staining; and 3, strong staining. According to the percentage of positive-staining cells in each microscopic field of view, the immunoreactivity scores ranged as follows: 0, < 25%; 1, 25–50%; 2, 50–75%; and 3, 75–100%. A final score ranging from 0 to 9 was achieved by multiplying the intensity score by the immunoreactivity score. When the final scores were 0, 1–4, and ≥4, the expression levels were considered as negative, low, and high, respectively.

### 2.4 Statistical analysis

All statistical analyses were performed using the Graph Pad software (Graph Pad version 7.0). The differences in the CBX7, E-cad, and VIM expression levels between the CC tissues and non-tumorous cervical tissues, and the difference of CBX7 expression level in clinicopathological parameters were analyzed by the *χ* ^2^ test. Partitions of *χ* ^2^ method were used for pairwise comparison of multiple groups. Correlation of the CBX7 level with the E-cad and VIM levels was analyzed using the Kappa correlation. The OS was measured by the Kaplan–Meier method and the difference in survival was analyzed by the Log-rank test. Pairwise comparisons between group levels were calculated with corrections for multiple testing. The prognostic values of these ten variables were tested by the univariate Cox analysis. The statistically significant prognostic factors (*P* < 0.05) in the univariate Cox analysis were further analyzed by the multivariate Cox proportional hazard models. All statistical tests were two-sided. *P* < 0.05 was considered statistically significant.

## 3 Results

### 3.1 CBX7 is related to proliferation activity of CC tissues

The CBX7 expression levels in the CC tissues were detected with the IHC staining, and the relationship of CBX7 expression with clinicopathological variables was analyzed. The CBX7 expression was divided into negative expression, low expression, and high expression. Our results showed that CBX7 expression was significantly different among patients with different clinical stages (*P* < 0.05), histological differentiation (*P* < 0.001), lymphatic metastasis (*P* < 0.001), and vascular invasion (*P* < 0.001) (Table 1). The pairwise comparison results of partitions of the *χ* ^2^ method show that there was no statistical difference in the negative expression rate and low expression rate among patients with different stages (*P* > 0.05). The patients of II, III–IV stages had lower number of CBX7 high expression than stage I patients (*P* < 0.05). The number of poorly differentiated patients with CBX7 negative expression patients was more than that of moderately differentiated and well-differentiated patients, and the number of poorly differentiated and moderately differentiated patients with high expression was less than that of well-differentiated patients (*P* < 0.05). The patients with lymphatic metastasis and vascular invasion had higher proportion of CBX7 negative expression but lower proportion of CBX7 high expression (*P* < 0.05). These results suggest that CBX7 is associated with the progression of CC.

**Table 1:** Expression of CBX7 in CC tissue and its association with clinicopathological features

		*N*	CBX7			
			Negative	Low	High	*P*
Stratified age	<40 years	74	22	27	25	0.750
	≥40 years	46	14	14	18	
Histology	Squamous	65	22	20	23	0.200
	Adenocarcinoma	29	9	15	5	
	Adenosquamous	26	5	6	15	
Clinical stages (FIGO)	Stage I	32	6^a^	7^a^	19^a^	0.031^*^
	Stage II	48	16^a^	19^a^	13^b^	
	Stages III–IV	40	13^a^	16^a^	11^b^	
Histological differentiation	High	45	7^a^	10^a^	28^a^	0.000^**^
	Moderate	43	7^a^	28^b^	8^b^	
	Low	32	22^b^	3^a^	7^b^	
Tumor diameter (mean, SD)	<4 cm	69	23	19	27	0.968
	≥4 cm	51	13	22	16	
Lymphatic metastasis	Absent	75	13^a^	26^a^	36^a^	0.000^**^
	Present	45	23^b^	15^a^	7^b^	
Vascular invasion	Absent	85	16^a^	33^a^	36^a^	0.000^**^
	Present	35	20^b^	8^a^	7^b^	
Fertility	No	13	4	4	5	0.925
	Yes	107	32	37	38	
Menopause	No	44	14	12	18	0.717
	Yes	76	22	29	25	

Note: Partitions of *χ* ^2^ method were used for pairwise comparison of multiple groups. When the columns are marked with the same superscript letter (a or b), it means that the two columns are not statistically significant; when the columns are marked with different superscript letters (a and b), it means that the comparison between the two columns is statistically significant. *P < 0.05 and **P < 0.001.

### 3.2 CBX7 expression is positively correlated with survival of CC cases

**Figure 1:**
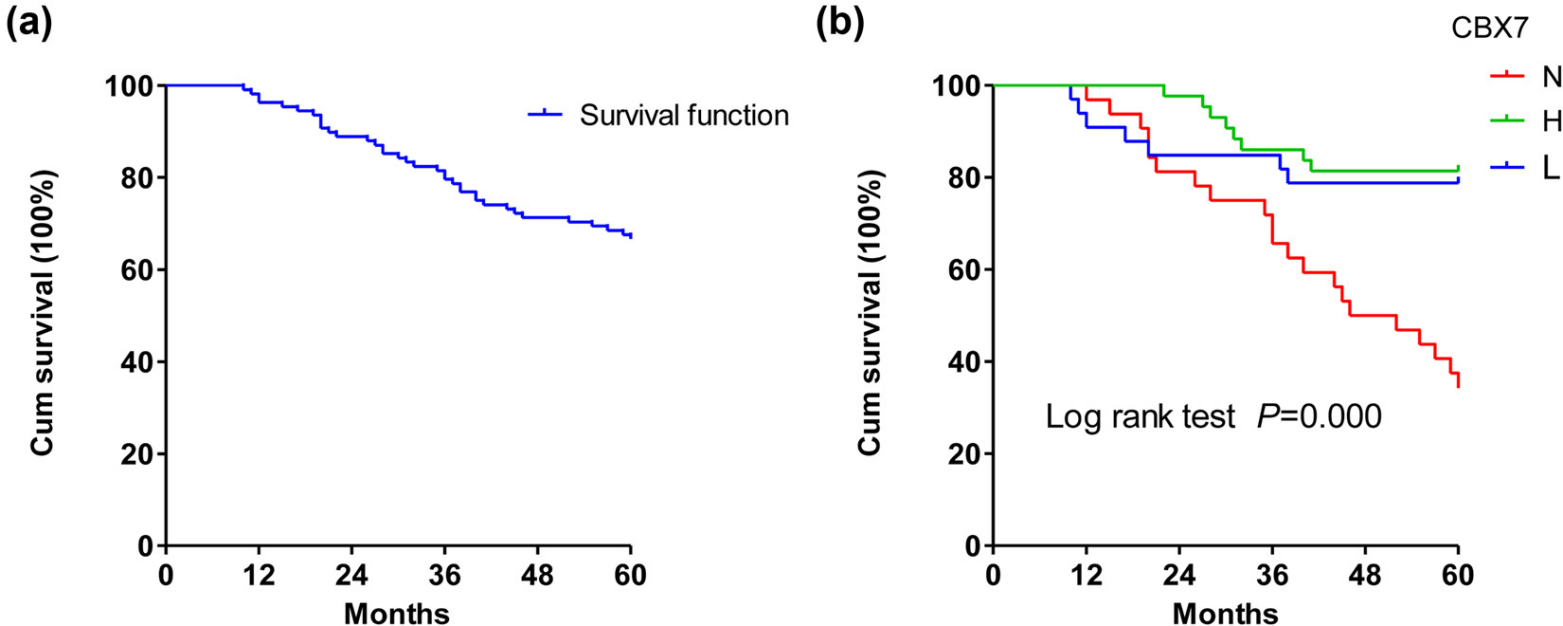
Survival curves of patients with CC. (a) OS curve. (b) Survival of patients with tumors lacking (N) or expressing low (L) or high (H) levels of CBX7.

The correlation of CBX7 expression with the survival of CC cases was investigated next. Our results showed that the median follow-up was 60 months. At the last follow-up, among the 120 CC patients, 72 (60.0%) patients were alive, 37 (30.8%) patients died of CC, and 11 (9.2%) patients were lost or died because of other reasons. Thereafter, the well-known clinicopathological prognostic parameters in CC were analyzed. The univariate Cox regression analysis showed that the clinical stages (*P* < 0.01), histological differentiation (*P* < 0.01), lymphatic metastasis (*P* < 0.01), vascular invasion (*P* < 0.01), and expression intensity of CBX7 (*P* < 0.01) were prognostic factors significantly associated with the OS (Table 2). Kaplan–Meier curves were used to analyze the 5-year OS rate of patients with different CBX7 expression levels (Figure 1). The survival rate of CBX7 with different expression levels was statistically significant (Log-rank test, *P* < 0.001). Pairwise comparisons showed that the survival rate of patients with negative CBX7 (11/36, 30.56%) was significantly shorter than that of patients with low expression (26/41, 63.41%) (*P* < 0.001) or high expression (35/43, 81.40%) (*P* < 0.001). However, there was no statistically significant difference in survival between patients with low and high CBX7 expression (*P* > 0.05), indicating that the absence of CBX7 expression predicts a poor OS in patients with CC. However, the cofounders were not adjusted in Kaplan–Meier curves.

**Table 2:** Cox regression analysis of prognostic factors of OS

Clinicopathological features	Univariate Cox regression		Multivariate Cox regression	
	HR (95% CI)	*P*	HR (95% CI)	*P*
Age, median (IQR)	0.919 (0.974–0.600)	0.915		
**Histology, No. (100%)**				
Squamous	1			
Adenocarcinoma	1.334 (0.652–2.730)	0.430		
Aden squamous	0.576 (0.216–1.537)	0.271		
**Clinical stages, FIGO, No. (100%)**				
I	1		1	
II	1.625 (0.673–3.922)	0.280	0.784 (0.347–1.784)	0.566
III–IV	3.435 (1.467–8.045)	0.004**	1.069 (0.443–2.581)	0.882
**Histological differentiation, No. (100%)**				
High	1		1	
Moderate	1.282 (0.456–3.600)	0.638	1.393 (0.475–4.081)	0.546
Low	8.093 (3.250–20.155)	0.000***	4.662 (1.695–12.820)	0.003**
**Tumor diameter, No. (100%)**				
<4 cm	1			
≥4 cm	1.057 (0.375–2.985)	0.916		
**Lymphatic invasion, No.(100%)**				
Absent	1		1	
Present	9.319 (2.236–38.756)	0.002**	4.276 (0.847–21.581)	0.079
**Vascular invasion, No. (100%)**				
Absent	1		1	
Present	7.417 (3.607–15.262)	0.000***	2.943 (1.250–6.928)	0.013*
**CBX7 expression, No. (100%)**				
Negative	1		1	
Low	0.283 (0.134–0.600)	0.001**	0.388 (0.162–0.930)	0.034*
High	0.118 (0.045–0.313)	0.000***	0.318 (0.108–0.941)	0.038*
**Fertility, No. (100%)**				
No	1			
Yes	1.635 (0.791–3.378)	0.184		
**Menopause, No. (100%)**				
No	1			
Yes	0.744 (0.290–1.911)	0.539		

Note: HR, hazard ratio.*P < 0.05, **P < 0.01, and ***P < 0.001.

To further determine the relationship between the survival and the clinicopathological features for CC patients, the multivariate Cox regression model adjusted for statistically significant prognostic factors was used. The clinicopathological features with statistical significance in the univariate analysis (clinical stage, histologic differentiation, lymphatic metastasis, vascular invasion, and CBX7 expression) were examined in the multivariate analysis (Table 2). Our results showed that the risk of death was 4.622 times higher in poorly differentiated patients than in highly differentiated patients (hazard ratio [HR], 4.622; 95% CI, 1.695–12.820). The risk of death in patients with lymphatic metastasis was 2.943 times higher than that in patients without vascular invasion (HR, 2.943; 95% CI, 1.25–6.928; *P* < 0.01). The risks of death in patients with low and high CBX7 expression were 0.388 times and 0.318 times of those with negative expression, respectively (HR, 0.388; 95% CI, 0.162–0.930; *P* < 0.05; HR, 0.318; 95% CI, 0.108–0.941; *P* < 0.05) (Table 2). The low expression of CBX7 indicates the disease severity and predicts poor disease prognosis.

### 3.3 Expression levels of CBX7 and E-cad are lower, whereas that of VIM is higher in CC tissues

**Figure 2:**
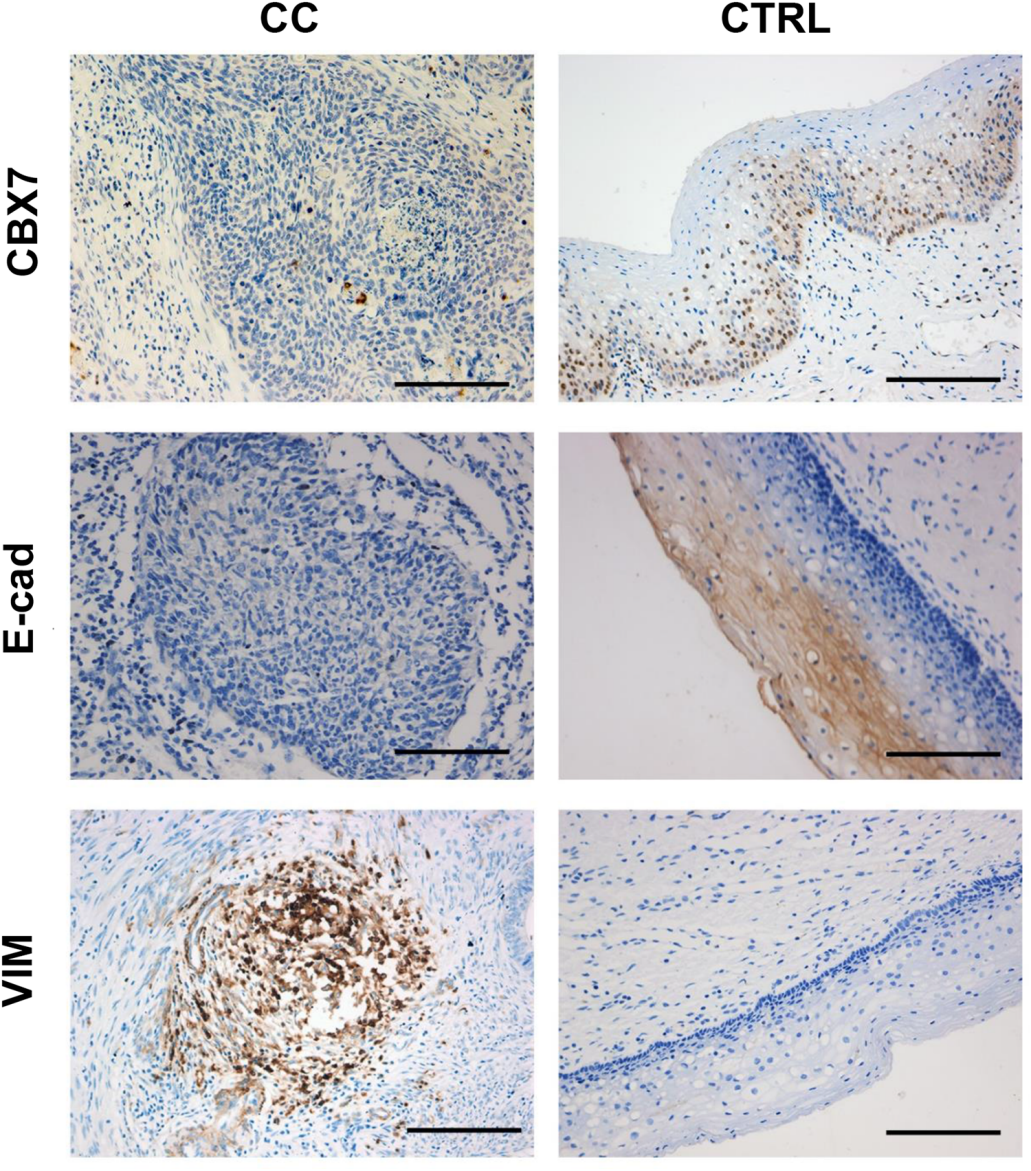
IHC staining. IHC staining was performed to detect the expression of CBX7, E-cad, and VIM in the CC and adjacent normal cervical tissue samples. Scale bar, 10 mm.

The expression levels of CBX7, E-cad, and VIM in the CC tissues and the matching adjacent non-tumorous cervical tissues were detected with the IHC staining. Our results showed that CBX7 was expressed on the cell nucleus, E-cad was expressed on the cell membrane, and VIM was expressed in cytoplasm (Figure 2). Moreover, the CBX7, E-cad, and VIM expression levels in the CC tissues and non-tumorous tissues were significantly different (Table 3). The Chi-square results showed that, compared with cancer tissues, the expression levels of CBX7 and E-cad negative in adjacent non-tumor tissues were lower (*P* < 0.05) and that of VIM was higher. There was no statistically significant difference in the low CBX7 expression between cancer and adjacent tissues (*P* > 0.05). The high expression of CBX7 and the low and high expression levels of E-cad were higher in the adjacent non-tumor tissues, whereas the low and high expression levels of VIM were lower (*P* < 0.05).

**Table 3:** Expression of CBX7, E-cad, and VIM in CC and adjacent non-tumorous cervical tissues

	CBX7 (*N*, %)			E-cad (*N*, %)			VIM (*N*, %)		
	Negative	Low	High	Negative	Low	High	Negative	Low	High
CC	36 (30.0)	41 (34.2)	43 (35.8)	89 (74.2)	16 (13.3)	15 (12.5)	27 (22.5)	43 (35.8)	50 (41.7)
Control	19 (15.8)	42 (35.0)	59 (49.2)	8 (6.6)	58 (48.3)	54 (45.0)	102 (85.0)	8 (6.7)	10 (8.3)
*χ* ^2^		7.776			115.520			94.291	
*P*		0.020			0.000			0.000	

Note: Partitions of *χ* ^2^ method were used for pairwise comparison of multiple groups.

### 3.4 Expression of CBX7 is positively correlated with E-cad and negatively correlated with VIM

To prove the mechanism concerning the metastasis and invasion of CC with CBX7 was related to EMT, the association between the expression levels of CBX with E-cad and VIM in the CC tissues was demonstrated. Our results showed that the CBX7 expression was positively correlated with the E-cad expression (Kappa = 0.236; *P* < 0.001; Table 4) and negatively correlated with the VIM expression (Kappa = −0.231; *P* < 0.001; Table 5).

**Table 4:** Correlation between CBX7 and E-cad

CBX7	E-cad Freq (%)			Total
	Negative	Low	High	
Negative	32 (26.7)	2 (1.7)	2 (1.7)	36
Low	27 (22.5)	13 (10.8)	1 (0.8)	41
High	30 (25.0)	1 (0.8)	12 (10.0)	43
Total	89	16	15	120
Kappa	0.236			
*P*	0.000			

**Table 5:** Correlation between CBX7 and VIM

CBX7	VIM Freq (%)			Total
	Negative	Low	High	
Negative	3 (2.5)	5 (4.2)	28 (23.3)	36
Low	2 (1.7)	19 (15.8)	20 (16.7)	41
High	22 (18.3)	19 (15.8)	2 (1.7)	43
Total	27	43	50	
Kappa	−0.211			
*P*	0.001			

## 4 Discussion

CBX7 is one of the Polycomb group complexes, which can function independently in the initiation and progression of various cancers [13,14,15]. In a previous study, the CBX7 overexpression has been associated with the poor prognosis of the ovarian adenocarcinomas through inhibiting the TRAIL-induced apoptotic pathway, suggesting the carcinogenic effects of CBX7 [8]. Other studies have shown that the low CBX7 expression was an independent predictor for poor prognosis in colon cancers, and the CBX7 negatively regulates KRAS through miR-155 to inhibit the anti-apoptotic activity, suggesting that CBX7 may play a role as an antioncogene [6,16]. Therefore, it has been speculated that the function of CBX7 might be influenced by the cellular environment, which may vary from cell to cell. To explore the role of CBX7 in CC, the correlation between CBX7 and clinicopathological features was analyzed herein. Our results showed that the negative expression of CBX7 was positively associated with poor clinical outcomes, such as lymphatic metastasis. Moreover, the 5-year OS of patients with no CBX7 expression was significantly worse than the patients with low or high CBX7 expression. The 5-year OS rate of patients with negative expression of CBX7 was lower than that reported by the American Cancer Society (66% for CC) [17]. The univariate survival and multivariate Cox regression for the OS analysis revealed that the CBX7 expression was noted for the independent prognostic significance. In line with the results of CBX7 in the glioma and pancreatic cancers [18,19], the low CBX7 expression was closely related to the poor disease prognosis of CC. A previous study has shown that comparing the minimally invasive surgery (robot-assisted or laparoscopic) with open surgery, CC shows a lower disease-free survival and OS rates [20]. Preoperative biopsy and tumor size >2 cm are possibly surgery-related factors associated with poorer oncologic outcomes in patients who underwent laparoscopic surgery for apparent early stage CC [21]. Given the number of factors have not been taken into account, and the Kaplan–Meier curves were not adjusted for confounders in this study, it is necessary to include more independent variables to construct a clinical prognosis model to predict that the survival rates of patients with CC at different time after surgery and independent prognostic risk factors.

In this study, the molecular mechanism of CBX7 in the metastasis and invasion of CC was explored. Our results showed that the expression levels of CBX7 and E-cad in the CC tissues were lower than those in the adjacent non-tumorous tissues, whereas the expression level of VIM was higher. Moreover, CBX7 was positively correlated with E-cad, whereas negatively correlated with VIM. When the CBX7 is absent, the E-cad would also be decreased, whereas the VIM would also be increased. The loss of epithelial features and the acquisition of mesenchymal phenotype would lead to the increased abilities of cell metastasis and invasion, thus promoting the tumor progression [12,22]. Bao et al. [13] have shown that CBX7 is involved in the EMT in glioma cancer. Based on our results, the observed correlation may indicate that CBX7 is involved in the EMT. In order to further confirm the role of CBX7 in the occurrence of EMT, it is necessary to conduct a bioinformatics analysis to confirm whether the signaling pathways involved in CBX7 are associated with E-Cad and VIM and to detect the influence of different expressions of CBX7 on cell biological processes in CC.

In this study, based on our results, we concluded that low expression of CBX7 was involved in the occurrence of EMT in CC. There are several possible mechanisms that may be responsible for this association. In previous studies, low CBX7 expression may be involved in promoting the tumor metastasis, in which CBX7 binds to the histone deacetylase 2 and inhibits its activity, leading to the histone deacetylation in the E-cad promoter and finally increasing the E-cad expression [13,15]. The disrupted balance would induce damage to the E-cad surface and increase the cell metastasis, eventually leading to EMT and cancer progression [13]. In addition, HMGA1 can inhibit the high mobility group protein A1b (HMGA1b). HMGA1 exerts no oncogenic effects, and HMGA1 positively regulates the expression of SPPI. SPPI encodes the chemokine osteopontin, which plays an important role in the cancer progression [16] and promotes the tumor progression and metastasis, as an important regulator of EMT [23]. Therefore, the absence of CBX7 would negatively regulate the SPP1 gene expression through HMGA1b, thus reducing the E-cad, inducing the EMT, and eventually promoting the cancer progression. Further molecular biology studies are still needed to validate the aforementioned mechanisms.

This study has some limitations. For example, the main viewpoints of this study are based on the limited sample size, and these samples were from one province. This study may have limited generalizability.

In conclusion, our results showed that the absence of CBX7 indicated a lower differentiation phenotype and poor prognosis. The expression of CBX7 may serve as a valuable tool in the clinical assessment of tumor biological behavior and disease prognosis in patients with CC.
